# Emerging concepts in the pathogenesis of the *Streptococcus pneumoniae*: From nasopharyngeal colonizer to intracellular pathogen

**DOI:** 10.1111/cmi.13077

**Published:** 2019-07-17

**Authors:** Karthik Subramanian, Birgitta Henriques‐Normark, Staffan Normark

**Affiliations:** ^1^ Department of Microbiology, Tumor and Cell Biology Karolinska Institutet Stockholm Sweden; ^2^ Clinical Microbiology Karolinska University Hospital Stockholm Sweden; ^3^ Lee Kong Chian School of Medicine (LKC) and Singapore Centre on Environmental Life Sciences Engineering (SCELSE) Nanyang Technological University Singapore

**Keywords:** immunity, intracellular survival, pathogenesis, persistence, pneumococci, *Streptococcus pneumoniae*

## Abstract

*Streptococcus pneumoniae* (the pneumococcus) is a human respiratory tract pathogen and a major cause of morbidity and mortality globally. Although the pneumococcus is a commensal bacterium that colonizes the nasopharynx, it also causes lethal diseases such as meningitis, sepsis, and pneumonia, especially in immunocompromised patients, in the elderly, and in young children. Due to the acquisition of antibiotic resistance and the emergence of nonvaccine serotypes, the pneumococcus has been classified as one of the priority pathogens for which new antibacterials are urgently required by the World Health Organization, 2017. Understanding molecular mechanisms behind the pathogenesis of pneumococcal infections and bacterial interactions within the host is crucial to developing novel therapeutics. Previously considered to be an extracellular pathogen, it is becoming evident that pneumococci may also occasionally establish intracellular niches within the body to escape immune surveillance and spread within the host. Intracellular survival within host cells also enables pneumococci to resist many antibiotics. Within the host cell, the bacteria exist in unique vacuoles, thereby avoiding degradation by the acidic lysosomes, and modulate the expression of its virulence genes to adapt to the intracellular environment. To invade and survive intracellularly, the pneumococcus utilizes a combination of virulence factors such as pneumolysin (PLY), pneumococcal surface protein A (PspA), pneumococcal adhesion and virulence protein B (PavB), the pilus‐1 adhesin RrgA, pyruvate oxidase (SpxB), and metalloprotease (ZmpB). In this review, we discuss recent findings showing the intracellular persistence of *Streptococcus pneumoniae* and its underlying mechanisms.

## 1 INTRODUCTION

*Streptococcus pneumoniae* is a Gram‐positive bacterium that colonizes the upper respiratory tract as a commensal in healthy individuals. This is called the “carriage” phase, which is usually asymptomatic and thought to be a prerequisite for a pneumococcal infection. However, when the pneumococcus migrates into the middle ear, the lungs, the blood stream, and the brain, it can cause common local diseases such as otitis media and potentially life‐threatening diseases such as pneumonia, septicaemia, and meningitis. Recently, pneumococci were also shown to cause microlesions in the myocardium that potentially contribute to the cardiovascular events that often follow an invasive pneumococcal disease (IPD), particularly in the elderly (Brown et al., 2014; Feldman, Normark, Henriques‐Normark, & Anderson, 2018). Pneumococci cause over one million deaths annually (Wahl et al., 2018). Treatment of pneumococcal infections can be complicated due to acquisition of resistance to commonly used antibiotics such as beta‐lactams, macrolides, and tetracyclins (Chenoweth, Saint, Martinez, Lynch, & Fendrick, 2000). Mortality rates in IPD were extremely high in the preantibiotic era, and the spread of pneumococcal resistance to antibiotics represents a serious public health treat worldwide. Therefore, the pneumococcus has been classified as a “priority” pathogen for which research on development of new antimicrobials is urgently required (World Health Organization, 2017).

Pneumococci are encapsulated bacteria that have a polysaccharide capsule surrounding the cell wall. The capsule is an important virulence factor, and currently, there are around 100 different capsular serotypes identified (Geno et al., 2015). Based on the most prevalent serotypes causing IPD, two types of pneumococcal vaccines are being used. First, the nonconjugated 23‐valent pneumococcal polysaccharide vaccine was launched, and more recently, conjugated polysaccharide vaccines (PCVs) were developed to provide a better protective effect especially in the risk groups such as the small children. PCVs have been introduced in childhood vaccination programs in many countries, and this has resulted in significant decreases in the number of IPD cases among vaccinated children. PCVs have also reduced nasopharyngeal colonisation of vaccine‐type (VT) strains in healthy children (Lindstrand et al., 2016). However, in some studies, PCV vaccination has not decreased pneumococcal carriage rates in children due to replacement of VT with nonvaccine‐type (NVT) strains (Galanis et al., 2016). This profound serotype replacement in the carrier population has led to an expansion of NVTs among IPD cases, even in nonvaccinated age groups. In the elderly, the incidence of IPD has therefore only decreased moderately in the post‐PCV era (Hanquet et al., 2019; Naucler et al., 2017), calling for novel approaches to develop protective vaccines or other intervention approaches for the elderly, for example, based on conserved virulence proteins, rather than capsular polysaccharides. Such approaches require a deeper understanding on how various virulence proteins contribute to colonisation, invasion, and evasion of host‐clearing mechanisms.

The pneumococcus expresses several virulence factors, such as the pore‐forming toxin, pneumolysin (PLY), adhesins (pneumococcal adhesion and virulence protein A [PavA] and pneumococcal adhesion and virulence protein B [PavB]), pili, pyruvate oxidase (SpxB), IgA1 protease, and exoglycosidases, to promote its survival in the host. The dynamics and mechanisms underlying the transition of the pneumococcus from colonisation to disease remain to be fully understood. In the nasopharynx, the population of colonising bacteria are controlled by the host inflammatory response orchestrated by neutrophils and macrophages. To establish an infection, pneumococci need to translocate from the nasopharynx to internal organs such as the lungs, middle ear, spleen, bloodstream, brain, or heart. Pneumococci have been shown to transmit themselves to new hosts via PLY‐mediated enhancement of bacterial shedding in nasal secretions (Zafar, Wang, Hamaguchi, & Weiser, 2017). To enable successful transmission to new hosts, the pneumococcus needs to maintain its survival without killing the colonising host. The focus of this review is to discuss recent findings in the field that identify intracellular survival of pneumococci in various organs and mechanisms for bacterial invasion of host cells.

## 2 NASOPHARYNGEAL COLONISATION

Nasopharyngeal colonisation is the first step in the life cycle of the pneumococcus in the host and usually precedes progression to invasive disease. During colonisation, pneumococci encounter immune defences from the host as well as competition with other members of the nasopharyngeal flora. Major steps in establishment of carriage are evasion of mucosal clearance and adhesion to the epithelium. To aid its colonisation, the pneumococcus expresses several virulence factors that are summarised in Table 1. The usually negatively charged capsule repels the mucosal polysaccharides, and the adhesion proteins, PavA, PavB, and enolase (Eno), bind to the extracellular matrix proteins, fibronectin and plasminogen (Bergmann et al., 2001; Holmes et al., 2001). Furthermore, the pneumococcal pilus‐1 mediates adhesion to epithelial cells also in encapsulated bacteria due to its polymeric nature and the presence of the pilus‐associated RrgA adhesin (Amerighi et al., 2016; Barocchi et al., 2006). To avoid clearance by the mucus, pneumococci utilize the matrix metalloprotease ZmpA, which cleaves mucosal IgA to evade complement activation, as well as clearance by the mucociliary flow (Roche, Richard, Rahkola, Janoff, & Weiser, 2015). The pneumococcus also expresses exoglycosidases such as neuraminidase (NanA), β‐galactosidase (BgaA), and β‐*N*‐acetylglucosaminidase (StrH) that deglycosylate host glycoproteins, releasing sugars as a nutrient source and exposing hidden receptors for adhesion to the epithelium (King et al., 2006). The pneumococcal surface proteins PspA, CbpA (PspC), and Pht block complement deposition. To outcompete the other cocolonising bacteria, the pneumococcus produces bacteriocins called pneumocins that mediate intraspecific competition (Dawid et al., 2007; Miller, Abrudan, Roberts, & Rozen, 2016). Thus, several pneumococcal virulence factors aid its successful colonisation of the nasopharynx (Table 1).

**Table 1:** Role of pneumococcal virulence factors in nasopharyngeal colonisation

Virulence factor(s)	Role in pathogenesis	Reference(s)
Polysaccharide capsule	Avoid entrapment in the mucus	Nelson et al. (2007)
Peptidoglycan‐*N*‐acetylglucosamine deacetylase (PgdA), Adr, an *O*‐acetyl transferase	Modify peptidoglycan to confer resistance to mucosal lysozyme	Davis, Akinbi, Standish, and Weiser (2008)
Exoglycosidases—neuraminidase A (NanA), β‐galactosidase (BgaA), and StrH EndoD	Degrade mucus and releases sugars from host glycoproteins; exposes host receptors for pneumococcal adhesion	Uchiyama et al. (2009); Limoli, Sladek, Fuller, Singh, and King ( 2011 ) ; and King, Hippe, and Weiser (2006)
Pneumococcal adherence and virulence proteins—PavA, PavB, enolase (Eno), foldase protein, PrsA, Peptidyl‐prolyl isomerases—SlrA and PpmA Pneumolysin (PLY) Pilus‐1 RrgA	Promote adhesion to endothelium and/or epithelium	Holmes et al. (2001), Bergmann, Rohde, Chhatwal, and Hammerschmidt (2001), Jensch et al. (2010), Cron et al. (2009), Hermans et al. (2006), Rubins et al. (1998), Barocchi et al. (2006), Amerighi et al. (2016), and Nelson et al. (2007)
Capsule Pneumococcal surface protein A (PspA) Choline‐binding protein A (CbpA/PspC) Pneumococcal histidine triad protein (Pht) Pneumolysin (PLY) Endopeptidase PepO	Complement evasion	Hyams, Camberlein, Cohen, Bax, and Brown (2010); Tu, Fulgham, McCrory, Briles, and Szalai (1999); Paton, Rowan‐Kelly, and Ferrante (1984), and Paton et al. (1984)
piuA, piaA and pitA (Fe ^2+^ ) psaA (Mn ^2+^ ) adcA and adcAII (Zn ^2+^ )	Acquire metal ions from the host	Paton et al. ( 1984 ) , McAllister et al. ( 2004 ) , and Plumptre et al. (2014)
Bacteriocins (pneumocins)—blpM and blpN Pneumocyclicin	Mediate intraspecies competition with other nasopharyngeal microbiota	Dawid, Roche, and Weiser (2007) and Bogaardt, van Tonder, and Brueggemann (2015)

To cause disease, the pneumococcus needs to migrate from the nasopharynx to invade organs such as the lungs and reach other internal organs via the blood. Studies show that coinfections with viruses increase pneumococcal carriage density and facilitate aspiration into the lungs (McCullers, 2014; Nakamura, Davis, & Weiser, 2011). Viral‐induced nasal inflammation causes loss of epithelial integrity, increased secretion of proinflammatory cytokines, and higher nutrient availability, thereby promoting pneumococcal growth and chances of its transmission into the lungs. To establish stable colonisation, pneumococci need to evade host immune responses. Pneumococcal colonisation induces influx of macrophages into the nasal lumen, and clearance is mediated by Toll‐like receptor‐dependent responses (Zhang, Clarke, & Weiser, 2009). To avoid clearance by the host, the pneumococcus has been shown to drive an immunoregulatory response characterised by higher levels of the transforming growth factor β (TGF‐β) and T‐regulatory (Treg) cells (Neill et al., 2014). This is supported by the clinical finding that Th17:Treg ratio was significantly higher in carriage‐negative than in carriage‐positive children (Mubarak et al., 2016). A recent study found that the mannose receptor C‐type lectin 1 (MRC‐1)‐expressing macrophages in the nasopharynx promote pneumococcal airway colonisation by inducing an inflammatory response driven by IL‐10 and TGF‐β (Subramanian et al., 2019). Interaction with MRC‐1 was mediated by the pneumococcal toxin PLY, and colonisation was impaired in MRC‐1 knockout mice. Hence, the pneumococcus modulates the local inflammatory response in the airways to establish colonisation. In the upcoming sections, we discuss recent literature identifying pneumococcal invasion into different organs and mechanisms that promote intracellular survival.

## 3 INTRACELLULAR SURVIVAL WITHIN MRC‐1^+^ MACROPHAGES AND DENDRITIC CELLS IN THE LUNGS

Pneumonia occurs when bacteria spread from the nasopharyngeal niche in the upper airways into the alveolar air space of the lungs. The pneumococcal polysaccharide capsule and the cytolytic toxin PLY have been shown to be indispensable for prolonged colonisation and invasive disease (Kadioglu et al., 2002; Kadioglu, Weiser, Paton, & Andrew, 2008). In the lungs, resident macrophages and dendritic cells (DCs) constitute the first line of immune defence against airborne pathogens including pneumococci. DCs found in the proximity to alveolar epithelial cells inhale bacterial pathogens to activate T‐ and B‐cell responses (Holt, 2005). Macrophages are a key component of the antimicrobial defence, but their role in pneumococcal infections is complex. Alveolar macrophages play a direct antimicrobial role through their ability to phagocytose and kill pneumococci (Jonsson, Musher, Chapman, Goree, & Lawrence, 1985), but they also have immunomodulatory functions and limit inflammation via removal of dead or dying neutrophils in the lungs during pneumococcal pneumonia (Knapp et al., 2003). Moreover, a study showed that expression of the pilus‐1 adhesin RrgA increases uptake of pneumococci into murine and mice macrophages, which was dependent on the presence of complement receptor 3 (Orrskog et al., 2012)

Although macrophages accumulate in the nasopharynx and draining lymph nodes during prolonged pneumococcal carriage in mice, this does not result in clearance of bacterial colonisation (Neill et al., 2014). The long‐standing question in the field is, How does the pneumococcus overcome the immune response in the airways to promote nasopharyngeal colonisation or pneumonia? Further, it is not known which receptors recognize the pneumococcus in the low‐opsonic environment of the nasopharynx, although a previous study showed that the mannose receptor (MRC‐1) can bind to some polysaccharide capsules of *S. pneumoniae* in vitro (Zamze et al., 2002).

Littmann et al. (2009) showed that wild‐type PLY‐expressing pneumococci inhibit human DC maturation, cytokine and inflammasome activation, and that these PLY‐expressing bacteria are localised in different intracellular compartments compared with an isogenic PLY mutant. The cytokine inhibition in DCs was not due to PLY‐mediated cell death because it also occurred at the low multiplicity of infection (MOI) of 1, when the cells were viable. Although this study demonstrates enhanced uptake of PLY‐expressing pneumococci, it did not identify the host receptor that binds to PLY and mechanisms behind the differential intracellular localisation of the wild‐type and PLY mutant.

Subramanian et al. (2019) investigated the inflammatory response and Toll‐like receptor signalling in different immune cells upon infection with a wild‐type and PLY mutant strain. The authors found that PLY inhibited cytokine responses in primary human DCs and in murine alveolar macrophages, whereas the pattern was reverse in neutrophils and monocyte‐derived macrophages (Subramanian et al., 2019). Previous studies had reported a predominantly inflammatory role of PLY (McNeela et al., 2010; Zafar et al., 2017), but they did not investigate different infection doses and cell types. However, the infection dose is critical because low‐dose pneumococcal carriage induces immunoregulatory responses, characterised by a sustained elevation of nasopharyngeal TGF‐β1, regulatory T cells, and MRC‐1‐expressing macrophages in contrast to a high‐density infection (Neill et al., 2014). Subramanian et al. compared different MOIs and found that at a low MOI of 1 or below, the PLY mutant strain D39Δ*ply* induced higher IL‐1β release compared with wild‐type bacteria (Subramanian et al., 2019). At a higher MOI of 10, the cytokine pattern was reversed, but the cell death was higher. Transcriptomic analysis revealed that PLY induced a global repression of inflammatory cytokines and chemokine genes in DCs. The anti‐inflammatory response of PLY was specifically observed in MRC‐1‐expressing DCs and alveolar macrophages in the lungs, indicating that these are the predominant intracellular niches for pneumococci in the lungs. Therefore, the infection dose and cell type are important factors that determine host responses to pneumococcal infections.

For several decades, PLY was only thought to bind to cholesterol on the host cells, and binding to a host receptor was not demonstrated. However, this dogma was challenged by the finding that PLY binds to MRC‐1 on DCs and alveolar macrophages in the lungs (Subramanian et al., 2019). MRC‐1 (also called CD206) is a classical marker for alternatively activated macrophages and a phagocytic receptor that is mostly expressed by tissue macrophages, including alveolar macrophages in the lungs. MRC‐1 binds to both endogenous and microbial antigens such as some capsular polysaccharides of *S. pneumoniae* (Linehan, Martinez‐Pomares, da Silva, & Gordon, 2001; Zamze et al., 2002). MRC‐1 mediated uptake of wild‐type, but not of PLY‐deficient, pneumococci, into DCs and macrophages, and internalised pneumococci in MRC‐1‐coated vacuoles did not fuse with acidic lysosomes. It was therefore proposed that the pneumococcus utilizes MRC‐1‐mediated uptake to persist intracellularly within DCs and macrophages in the lungs and utilizes them as Trojan horse to spread the infection (Subramanian et al., 2019). During this persistence phase, pneumococci suppress the immune response by inducing negative regulatory molecules such as suppressor of cytokine signalling and FoxP3^+^ immunoregulatory T cells. In mice, intranasal administration of antibodies targeting MRC‐1 reduced the bacterial load in the lungs and restored the inflammatory cytokine response (Subramanian et al., 2019). MRC‐1 knockout mice showed significantly reduced pneumococcal carriage in the nasopharynx, indicating that this pathway could be important for colonisation as well. In conclusion, this study identified PLY to play a significant role in establishing residency within MRC‐1‐proficient immune cells, including DCs and alveolar macrophages, in the airways. Contrary to the notion that PLY is only released during bacterial autolysis, these results suggest surface exposure of PLY on intact bacteria. Other studies have shown evidence to suggest that PLY can be cell‐wall localised (Price & Camilli, 2009; Shak et al., 2013). PLY does not possess any signal peptide or known cell‐wall anchor motifs but has been shown to be enriched in extracellular vesicles formed from localised plasma membrane protrusions on the bacteria (Codemo et al., 2018). However, further studies are required to investigate the surface localisation of PLY and mechanisms underlying its membrane anchorage. Moreover, it remains to be shown whether intracellular pneumococci within the lungs could recolonize the nasopharynx, thereby facilitating transmission to new hosts.

## 4 REPLICATION WITHIN THE SPLEEN

The spleen is a major lymphatic organ that plays a vital role in the phagocytic clearance of pathogens from the bloodstream and production of opsonising antibodies. Clearance by the spleen accounts for the eclipse phase of a pneumococcal infection whereby bacterial numbers rapidly decrease in the bloodstream following intravenous inoculation. However, this raises the important question of how the pneumococcus causes septicemia despite clearance from the bloodstream by splenic macrophages. Ercoli et al. (2018) investigated this phenomenon and found that CD169^+^ splenic macrophages serve as a reservoir for intracellular replication of internalised pneumococci. Using a 1:1 inoculation of mice with D39 strains expressing green or red fluorescent proteins, they showed that bacterial foci within the spleen consisted entirely of single labelled bacteria. This supports the hypothesis that the infection likely originates from a single bacterium. Depending on the infection dose, they found that the pneumococcal clusters were almost exclusively located to the marginal sinus region of the spleen, specifically the CD169^+^ marginal zone metallophilic macrophages. CD169 (Siglec1/Sialoadhesin) is a lectin receptor that mediates uptake of sialylated bacteria and viruses by macrophages (Chang et al., 2014; Heikema et al., 2013; Klaas et al., 2012). These macrophages are known to be permissive for viral replication in mice and pigs due to their ineffective bactericidal mechanisms and higher expression levels of suppressors of cytokine signalling (Honke et al., 2011; Van Breedam, Verbeeck, Christiaens, Van Gorp, & Nauwynck, 2013). Using an ex vivo pig spleen perfusion model, they confirmed the role of CD169^+^ macrophages as a reservoir for intracellular replication of pneumococci within the spleen. Although this study convincingly demonstrated an intracellular niche for the pneumococcus within splenic macrophages that initiates septicemia, the exact subcellular localisation of pneumococci needs further clarification.

## 5 INVASION INTO THE HEART

Pneumococcal infections are major risk factors for cardiovascular diseases such as heart failure, and cardiac arrhythmia (Corrales‐Medina et al., 2012). Clinical studies reveal higher mortality in cardiac patients with underlying pneumococcal pneumonia (Musher, Rueda, Kaka, & Mapara, 2007). Pneumococci can invade the heart and form microlesions, thereby disrupting the electrophysiology and contractile function of cardiomyocytes (Brown et al., 2014). *S. pneumoniae* has been shown to be internalised by clathrin‐mediated endocytosis into the cardiomyocytes, wherein the pneumococci replicate within intracellular vacuoles (Brissac, Shenoy, Patterson, & Orihuela, 2018). Bacterial translocation into the heart tissue requires the pneumococcal adhesin CbpA (PspC) and the host receptors, laminin receptor and platelet‐activating factor receptor. The pneumococcal pore‐forming toxin PLY and pyruvate oxidase‐derived peroxide have been shown to play a major role in inducing cardiac failure by killing cardiomyocytes and infiltrating macrophages (Brissac et al., 2018; Gilley et al., 2016) . However, the intriguing question is, How does the pneumococcus establish itself within the myocardium without eliciting immune responses? Shenoy et al. (2017) showed by transmission electron microscopy that pneumococci replicate within cardiac lesions in unique intracellular vesicles of sizes 4–8 μm. Further, they showed that pneumococci formed biofilms within the heart and possessed a distinct transcriptomic profile when compared with blood‐isolated pneumococci. Key virulent genes that were specifically upregulated in heart‐isolated pneumococci include the adhesion proteins PavB and PspA, the capsular polysaccharide biosynthesis locus, metalloprotease ZmpB, SpxB, PLY, the autolysin LytA, and the choline‐binding protein PcpA. By comparing biofilm‐producing pneumococci (TIGR4) with the planktonic controls, they showed that biofilm formation enables the pneumococcus to subvert cytokine production by macrophages and neutrophil influx. This was attributed to PLY‐mediated killing of immune cells and was more pronounced in the biofilm state than in planktonic cultures. However, the exact cell type(s) in the heart within which pneumococci replicate remains to be elucidated, although probable candidates include resident macrophages, cardiomyocytes, and fibroblasts.

## 6 TRAFFICKING ACROSS THE BLOOD–BRAIN BARRIER

*S. pneumoniae* is the major cause of bacterial meningitis in adults (Engelen‐Lee, Brouwer, Aronica, & van de Beek, 2016). Pneumococcal meningitis is associated with a high mortality, causing death in 18–30% of patients, and neurological sequalae such as hearing loss and cognitive disabilities occur in ~50% of survivors (Saez‐Llorens & McCracken, 2003; van de Beek, de Gans, Tunkel, & Wijdicks, 2006; Weisfelt et al., 2006). To cause meningitis, pneumococci in the bloodstream need to pass the blood–brain barrier (BBB), which is composed of brain microvascular endothelial cells. Recently, it was shown that by interactions between the pneumococcal pilus‐1 adhesin RrgA and the choline‐binding protein PspC, with two endothelial receptors, platelet endothelial cell adhesion molecule 1 and polymeric immunoglobulin receptor, pneumococci can invade the brain endothelium (Iovino et al., 2016; Iovino, Molema, & Bijlsma, 2014; Iovino, Seinen, Henriques‐Normark, & van Dijl, 2016). Ring, Weiser, and Tuomanen (1998) found that the transparent pneumococcal variant, characterised by lower capsule and higher CbpA expression, showed higher adhesion and transcytosis across brain microvascular endothelial cells via interaction with the platelet adhesion factor receptor. In contrast, the opaque colony variants were preferentially sorted to the lethal lysosomes. During transmigration across endothelial cells, pneumococci resided in intracellular vesicles although the exact mechanism of transmigration and nature of intracellular vesicles needs to be clarified. The main infection bottleneck for the bacteria is to escape lysosomal killing during their transcytosis across the endothelial cells. A recent study found that the pore‐forming toxin PLY is essential for pneumococci to thwart intracellular killing and trafficking across the BBB (Surve et al., 2018). They identified heterogenous expression of PLY within isogenic pneumococcal populations and different intracellular fates for the subpopulations. By comparing wild‐type pneumococci expressing high or low levels of PLY, they showed that the low‐PLY‐expressing subset showed higher intracellular survival within the brain endothelial cells by delaying autophagosomal maturation and proteasomal degradation. Moreover, the low‐PLY subset of pneumococci also had higher transcytosis efficiency in comparison with the high‐PLY subset. Using a bacteremia‐derived meningitis mouse model, they found that pneumococci in the bloodstream displayed heterogenous PLY expression, with the low‐PLY subset exhibiting higher trafficking efficiency into the brain (Surve et al., 2018). Comparison of PLY expression across different pneumococcal serotypes revealed lower surface expression by invasive serotype type 4, TIGR4, as compared with the serotype 2 strain D39, and the 19F strain A60. Collectively, this study showed that intracellular survival of pneumococci within brain endothelial cells facilitates transcytosis across the BBB to cause meningitis and suggests that spatio‐temporal regulation of PLY expression is key to pneumococcal invasion of the brain. This study opens new questions in the field of pneumococcal pathogenesis that need further investigation. Is stochastic regulation of PLY applicable in other facades of pneumococcal infection such as nasopharyngeal colonisation, pneumonia, and septicemia? Are other pneumococcal virulence factors such as the capsule also regulated heterogeneously? If so, could heterogeneity create bystander pneumococcal populations in vaccinated individuals that potentially could cause recurrent infections?

## 7 CONCLUSIONS AND OUTLOOK

**Figure 1:**
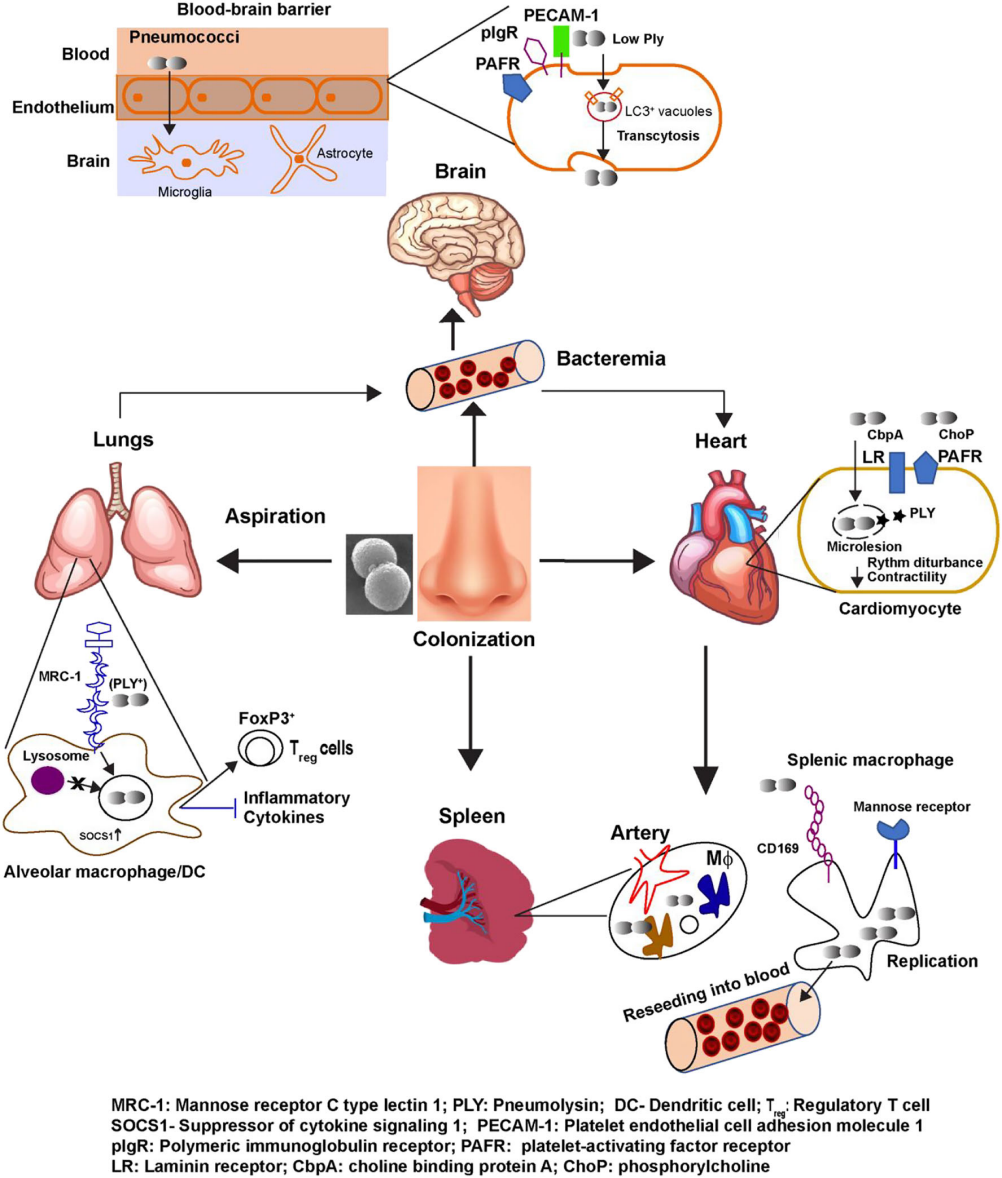
Model summarising mechanisms involved in pneumococcal invasion and intracellular survival within different organs. Nasopharyngeal colonisation is established upon inhalation of pneumococci and is a prerequisite for invasive disease as well as for transmission to new hosts. Dissemination of the pneumococcus into the lungs, heart, brain and spleen leads to diseases such as pneumonia, heart failure, meningitis, and bacteraemia. In the lungs, resident MRC‐1^+^ macrophages and DCs phagocytose pneumococci via interaction with PLY, and internalised pneumococci avoid lysosomal fusion and repress inflammatory cytokine production by upregulating the SOCS1. In the heart, the host receptors, PAFR and LR, promote pneumococcal invasion of cardiomyocytes wherein they form microlesions and inhibit contractibility and cardiac rhythm. In the brain, pneumococci interact with the endothelial receptors, PECAM‐1, pIgR, and PAFR, and transcytose across the endothelium to infect the underlying brain tissue. In the spleen, pneumococci utilize CD169 to infect splenic macrophages wherein they replicate intracellularly and reenter the bloodstream to cause sepsis

*S. pneumoniae* is an opportunistic member of the airway microbiota that has emerged as a top‐priority pathogen that is rapidly acquiring resistance to widely used antibiotics and evading current vaccine approaches. Although previously considered to be an extracellular pathogen, recent studies confirm that the pneumococcus can adopt an intracellular lifestyle. This minireview discusses the recent literature showing how pneumococci can invade and create intracellular niches within vital organs such as the lungs, heart, brain and spleen (summarised in Figure 1). Understanding the molecular events underlying the tissue invasion and intracellular survival is vital for the development of novel approaches to prevent and treat pneumococcal infections. Further studies are needed to investigate whether the intracellular niches constitute a “silent” persistent reservoir for the pathogen to reestablish colonisation and thereby transmit itself to new hosts. Moreover, detailed molecular and genetic characterisation of the intracellular variants of pneumococci by single‐cell high‐throughput sequencing would be useful to delineate which virulence factors are required to promote intracellular persistence. Novel therapeutic strategies targeting host receptors that have been shown to be involved in pneumococcal pathogenesis could be developed and employed in combination with current antibiotic therapy. One such approach would be to develop blocking antibodies against MRC‐1/Siglec 1 to counter bacterial invasion and replication in the lungs and spleen, respectively. In this regard, a recent study showed that inhibiting the brain endothelial receptors, polymeric immunoglobulin receptor and platelet endothelial cell adhesion molecule 1, reduces pneumococcal brain invasion in a bacterial meningitis model (Iovino et al., 2017). Identification of novel bacterial targets that promote intracellular persistence and immune evasion will contribute to the development of selective blockade of such pathways, as well as the development of novel vaccine candidates in the future.

## CONTRIBUTIONS

All the authors wrote, edited, and reviewed the manuscript.

## COMPETING INTERESTS

The authors declare no competing interests.
